# Minimal Clinically Important Difference (MCID) and Long-Term Prospective Validation of the AO Spine PROST (Patient Reported Outcome Spine Trauma)

**DOI:** 10.1177/21925682261445019

**Published:** 2026-04-18

**Authors:** Said Sadiqi, Charlotte Dandurand, Richard J. Bransford, Lorin M. Benneker, Andrei F Joaquim, Ulrich Spiegl, Jin W. Tee, Alexander R. Vaccaro, F. Cumhur Oner

**Affiliations:** 1 Department of Orthopaedics, 72507 St. Antonius Hospital, Utrecht, The Netherlands; 2 Combined Neurosurgical and Orthopedic Spine Program, Vancouver General Hospital, University of British Columbia, Vancouver, BC, Canada; 3 21618 Harborview Medical Center, University of Washington School of Medicine, Seattle, WA, USA; 4 Spine Unit, Sonnenhofspital Bern, University of Bern, Bern, Switzerland; 5 Neurosurgery Division, Department of Neurology, 28132 State University of Campinas, Campinas-Sao Paulo, Brazil; 6 Clinics of Trauma Surgery, Orthopaedics, and Reconstructive Surgery, München Klinik Harlaching, Munich, Germany; 7 Department of Neurosurgery, National Trauma Research Institute (NTRI), 5390 The Alfred Hospital, Melbourne, VIC, Australia; 8 Department of Orthopaedics, Rothman Institute, Thomas Jefferson University, Philadelphia, PA, USA; 9 Department of Orthopaedics, 8124 University Medical Center Utrecht, Utrecht, The Netherlands; 10 AO Innovation Translation Center, AO Foundation, Davos, Switzerland

**Keywords:** MCID, AO spine PROST, long-term, validity, spine trauma, HRQoL

## Abstract

**Study design:**

Prospective observational multicenter cohort study.

**Objectives:**

To determine Minimal Clinically Important Difference (MCID) of AO Spine PROST (Patient Reported Outcome Spine Trauma) and conducting a long-term prospective validation.

**Methods:**

Data were collected from a prospective observational international multicenter cohort study. Adults (18-65) with acute thoracolumbar (TL) burst fractures without neurologic deficits were enrolled, and followed for up to 2 years. Patients completed the AO Spine PROST, Oswestry Disability Index (ODI), EQ-5D, and Pain NRS. Characteristics were analyzed using descriptive statistics, MCID for PROST with distribution-based approach using the standard deviation (SD) of change in scores. Floor and ceiling effects were also evaluated. Internal consistency (Cronbach’s alpha, item-total correlation coefficient (ITCC) and pairwise Spearman correlation), construct validity (Pearson correlations (r_s_) with ODI, EQ-5D, Pain NRS), and responsiveness (effect sizes (ES) and standardized response mean (SRM)) were assessed.

**Results:**

Ninety-three patients were included. MCID for a moderate change (0.5*SD) in PROST score was 10.6. No floor or ceiling effects were observed. Internal consistency was high (Cronbach’s α = 0.9-1.0 and acceptable ITCC). PROST scores strongly correlated with ODI (rs = −0.67 to −0.89; *P* < .001), but correlations with EQ-5D were weak (r_s_ = −0.29 to 0.05; *P* > .005), except at 1-year follow-up. No consistent pattern was found with Pain NRS. Responsiveness was very good (ES = 3.2, SRM = 3.1; *P* < .001).

**Conclusions:**

The AO Spine PROST identified an MCID of 10.6 as indicative of a moderate clinically meaningful change. The instrument also showed strong internal consistency, construct validity, and excellent responsiveness in long-term follow-up.

## Introduction

The AO Spine PROST (Patient Reported Outcome Spine Trauma) was developed through a series of preparatory studies aimed at creating the first comprehensive patient-centered outcome measure designed for spine trauma.^1^ The development process followed the Core Set development methodology of the International Classification of Functioning, Disability and Health (ICF).^2^ During the preparatory phase of the project, 4 distinct studies were conducted. Three of these focused on identifying relevant ICF categories for assessing outcomes of traumatic spinal column injuries, drawing on perspectives from research, clinical experts, and patients.^3-5^ The fourth study evaluated different response scales to determine their suitability for inclusion in the questionnaire.^6^ The preparatory efforts were followed by a formal consensus process, that incorporated the earlier findings and expert feedback, ultimately resulting in the PROST—a 19-item scale based on 25 core ICF categories.^7^ Initial validation efforts established the PROST’s reliability and validity in both English and Dutch-speaking populations.^8,9^ Since then, the instrument has been translated into 18 languages, expanding its global applicability and supporting its use in diverse clinical settings.^10^ The PROST has also been increasingly adopted as an outcome measure in clinical research evaluating spine trauma interventions.

Despite these advancements, a critical psychometric property remains undefined: the Minimal Clinically Important Difference (MCID). The MCID represents the smallest change in score that patients perceive as beneficial, serving as a vital benchmark for evaluating treatment effects and guiding clinical decision-making.^11^ Without an established MCID, it remains challenging to distinguish statistically significant changes from those that are clinically relevant, which potentially limits the interpretability and practical application of PROST scores in both research and practice.

To address this gap, the present study aimed to determine the MCID for the AO Spine PROST using a distribution-based method. Additionally, a long-term prospective validation of the instrument’s psychometric performance was performed in an international patient cohort.

## Methods

### Study Procedures and Patients

This study utilized the data from a prospective observational international multicenter cohort study.^12,13^ It investigated the management of thoracolumbar burst fractures in neurologically intact patients aged 18 to 65 years with an acute (<10 days from injury) traumatic fracture, with or without a suspected Posterior Ligamentous Complex (PLC) injury, between T10 and L2. To ensure a relatively homogeneous cohort among spine trauma, patients with pathological fractures such as osteoporotic or neoplastic, prisoners, prior spinal surgery, multi-trauma with injury severity scores (ISS) greater than 16, and unable to understand or report outcomes were excluded. Patients were recruited from several hospitals worldwide participating in the trial entitled ‘Thoracolumbar burst fractures (AO Spine A3, A4 fractures) in neurologically intact patients: An observational, multicenter cohort study comparing surgical vs non-surgical treatment. (Spine TL A3/4 Study, ClinicalTrials.gov: NCT02827214).^14^ The 14 study sites represented North America (6 sites), Europe (5 sites), and 1 site each in India, Middle East and Australia. Each enrolling center obtained local approval from their local institutional review board (UBC CREB NUMBER: H16-02527). Treatment was not randomized but followed the standard clinical decision-making process in each institution and the judgment of the treating surgeon. Patients received either surgical stabilization or non-operative management, including orthosis, body cast, or no bracing. Written informed consent was obtained from all patients, and they were asked to complete the questionnaires at discharge (ie, baseline) and subsequently at 6 weeks, 3 months, 6 months, 1 year, and 2 years post-trauma.

### Instruments

Next to the AO Spine PROST, various other questionnaires were administered to the patients for validity purposes: Oswestry Disability Index (ODI), Pain NRS (Numeric Rating Scale) and EuroQol-5D (EQ-5D).^15,16^ The PROST is the first condition-specific patient-reported outcome measure (PROM) for spine trauma. It consists of 19 items covering a wide range of functional domains such as work/study, travelling, pain, urinating, sexual function, and emotional function.^10^ Each item is rated on a 0 to 100 numeric rating scale, with 0 indicating no functional at all and 100 the same level as pre-trauma, regardless of how well or poorly the patient priorly functioned. At the time of study initiation, only the Dutch and English versions of the PROST were available and validated, thus only participants fluent in English and Dutch were included.

The ODI is a widely used PROM designed to assess disability related to low back pain.^15^ It comprises 10 items spanning activities of daily living, each scored on a 6-point scale ranging from 0 (no disability) to 5 (maximum disability). The total score is expressed as a percentage, ranging from 0% (no disability) to 100% (completely disabled). Pain NRS is a widely used, unidimensional tool for assessing pain intensity. Patients rate their pain on an 11-point scale from 0 (no pain) to 10 (worst imaginable pain). The EQ-5D is a widely used generic instrument for evaluating health-related quality of life (HRQoL), with scores ranging from 0 to 1, where 1 represents optimal health.^17^ It comprises 2 components: the EQ-5D descriptive system and the EQ-5D visual analog scale (EQ VAS).

### Statistical Analysis

Patient characteristics were analyzed using descriptive statistics and including sample size (n), mean, standard deviation (SD), median, lower and upper values of the inter-quartile range, and minimum and maximum values. Categorical variables were summarized using the frequency and percentage for each category.

The MCID for the PROST was assessed using a distribution-based approach, assessing change in PROST scores between baseline and 1-year follow-up. First the standard deviation (SD) of change in PROST score between the aforementioned timepoints was calculated. The SD of this change was used to determine effect sizes according to Cohen’s criteria, with a small effect size defined as 0.2 × SD and a moderate effect size as 0.5 × SD. The MCID was calculated for the total group and also stratified by treatment modality (surgical vs. non-surgical) and fracture type (A3 vs. A4). The threshold of 0.5 SD was chosen in accordance with prior literature indicating that a moderate effect size corresponds to a clinically meaningful change.^18^

Floor and ceiling effects were also assessed, which would occur if >15% of the patients achieve the lowest or highest possible score, respectively.

Internal consistency was evaluated by Cronbach’s alpha, with a threshold of ≥0.7 considered acceptable.^19^ Additional reliability metrics included item-total correlation coefficients and pairwise Spearman correlations to examine the relationship between individual items and the overall scale. Item-total correlation coefficients below 0.2 were considered indicative of poor alignment with the total score and potential candidates for removal.^20^

Construct validity was assessed by examining both convergent and discriminant validity. Convergent validity refers to the extent to which the PROST correlates with other instruments as theoretically expected, while discriminant validity refers to a lack of correlation with constructs it is not expected to relate to.^21^ Pearson correlation coefficients were calculated between PROST scores and those of the ODI, Pain NRS, and EQ-5D. Correlations were examined at baseline and all follow-up time points, as well as for changes in scores between baseline and 1-year follow-up. For the ODI specifically, comparisons were limited to baseline, 3 months, 1 year, and 2 years, as data from other time points were not collected.

Responsiveness was evaluated by the effect size (ES) and the standardized response mean (SRM). ES was calculated as the mean change in score from baseline divided by the SD at baseline, while SRM was calculated using the mean change in score divided by the SD of the change score. Paired t-tests were used to assess the significance of change at the 1-year follow-up. According to established guidelines, both for ES and SRM values of 0.2-0.5 were interpreted as small, 0.5-0.8 as moderate, and ≥0.8 as large change.^19,21,22^

## Results

### Patient Characteristics

Out of 198 eligible and enrolled patients, 93 (47.0%) were either native or proficient speakers of English or Dutch, had a PROST score available at discharge or at any of the follow-up visit and were therefore included in the present analysis. Patient demographics are summarized in Table 1. In addition to the English- and Dutch-speaking sites in Australia, Canada, USA, and the Netherlands, also patients from non-English speaking sites were included, that is, Greece, India, and Switzerland. Patients could be included if they demonstrated sufficient proficiency in English as assessed by the local investigators. The cohort was predominantly male (57.0%) with a mean age of 41.4 years (SD 13.9; range: 18-65 years). A relatively small proportion were smokers (21.5%). Most participants were employed, self-employed, students, or homemakers (87.1%). The majority of injuries (73.1%) resulted from high-energy trauma. Surgical intervention was performed in 38.7% of cases. Further details regarding both surgical and non-surgical treatment approaches are provided in Table 2.

**Table 1.** Socio-Demographic and Clinical Characteristics of the Study Population (n = 93)

Male (%)	53 (57.0)
Age, mean ± SD (range) in years	41.4 ± 13.9 (18-65)
BMI, mean ± SD (range)	25.4 ± 4.5 (18.0-36.4)
Smoker (%)	20 (21.5)
Employment (%)	
Employed/self-employed/student/homemaker	81 (87.1)
Unemployed/retired	12 (12.9)
Charlson comorbidity index (%)	
0	86 (92.5)
≤4	7 (7.5)
>4	0 (0.0)
TLICS score, mean ± SD (range)	2.8 ± 1.2 (2.0-5.0)
PLC (%)	
Intact	64 (68.8)
Injury suspected/indeterminate	10 (10.8)
Injured	19 (20.4)
Additional spine injury (%)	21 (22.6)
Time from injury to treatment, mean ± SD (range) in days	2.3 ± 2.2 (0.0-10.0)
Trauma type (%)	
High energy trauma (eg, motor vehicle accident)	68 (73.1)
Low impact trauma (eg, falls at home)	25 (26.9)
Treatment (%)	
Surgical	36 (38.7)
Nonsurgical	57 (61.3)
Regions (%)	
Europe	42 (45.2)
North America	35 (37.6)
India	1 (1.1)
Middle East	0 (0.0)
Australia	15 (16.1)
Centers (%)	
Assiut, Egypt	0
Athens, Greece	5 (5.4)
Bern, Switzerland	14 (15.1)
Coimbatore, India	1 (1.1)
Iasi, Romania	0
Marbella, Spain	0
Melbourne, Australia	15 (16.1)
Morgantown, USA	8 (8.6)
Philadelphia, USA	6 (6.5)
Quebec, Canada	2 (2.2)
San Diego, USA	1 (1.1)
Syracuse, USA	2 (2.2)
Utrecht, The Netherlands	23 (24.7)
Vancouver, Canada	16 (17.2)

Abbreviations: PLC, posterior ligamentous complex; SD, standard deviation.

**Table 2.** Details on the Nonsurgical and Surgical Treatment

Nonsurgical treatment (n = 57)	
No immobilization (%)	27 (47.4)
Bed rest followed by immobilization with: (%)^a^	
Custom-molded or prefabricated total body contact thoracolumbosacral orthosis (TLSO)	19 (33.3)
Thermoplastic removable brace	0
Jewett hyperextension braces	8 (14.0)
Anterior hyperextension brace (ASH)	0
Taylor-knight brace	3 (5.3)
Plaster of Paris (POP)	0
Surgical treatment (n = 36)	
Duration of surgery, mean ± SD (range) in minutes	111.1 ± 64.3 (33.0-317.0)
Surgical subgroups (%)	
Open short segment posterior fixation	8 (22.2)
Open long segment posterior fixation	6 (16.7)
Percutaneous posterior fixation with or without vertebroplasty	14 (38.9)
Other treatment techniques	8 (22.2)
Bone substitutes (%)^a^	6 (16.7)
Autograft	2 (33.3)
Allograft	2 (33.3)
Matrices	3 (50.0)
Estimated blood loss, mean ± SD (range) in ml	271 ± 464 (0-2800)

Abbreviations: SD = standard deviation.

^a^Multiple answer options possible.

### MCID

As presented in Table 3, MCID values were calculated based on both small (0.2*SD) and moderate effect sizes (0.5*SD), based on the standard deviation of change in PROST scores from baseline to 1 year. For the overall cohort, the PROST score yielded an MCID of 4.2 for a small effect size and 10.6 for a moderate effect size. When stratified by treatment type, patients who underwent surgical management had a higher moderate effect size (13.5) compared to those treated non-operatively (8.0). Effect sizes were comparable between injury types, with A3 fractures showing a value of 10.2 and A4 fractures 11.1.

**Table 3.** Distribution-Based MCID Assessment for AO Spine PROST (0.2 and 0.5 × SD of Change)

	0.2*SD_diff_	0.5*SD_diff_
Total	4.2	10.6
Surgical treatment	5.4	13.5
Nonsurgical treatment	3.2	8.0
A3 type injury	4.1	10.2
A4 type injury	4.4	11.1

SD_diff_ = standard deviation of change in PROST score from baseline to 1 year.

### Floor and Ceiling Effects

Across all follow-up time points, fewer than 15% of participants achieved either the lowest or highest possible PROST scores. This indicates that neither floor nor ceiling effects were present, respectively, suggesting that the instrument has an appropriate scoring range for this patient population.

### Internal Consistency

The AO Spine PROST demonstrated excellent internal consistency, with Cronbach’s alpha values of 0.9 or higher at baseline and all follow-up time points (Table 4). Item-total correlation revealed that most of the items had sufficient correlation within the scale. Among the items with the lowest item-total correlations (0.30 or lower), ‘Urinating’ was most frequently flagged, followed by ‘Bowel movement’ and ‘Sleep’.

**Table 4.** Results for Internal Consistency (Cronbach’s α) of AO Spine PROST Across Different Timepoints

Timepoint	Cronbach’s α
Baseline	0.9
6-week	0.9
3-month	1.0
6-month	1.0
1-year	1.0
2-year	1.0

Cronbach α ≥ 0.7 indicate acceptable internal consistency, that is, the items are highly correlated and thus measure to high extent the same concept.

### Construct Validity

Overall, the correlation between the PROST and ODI was strong to very strong (r_s_ = −0.67 to −0.89) at each follow-up timepoint and when comparing baseline to 1-year follow-up (r_s_ = −0.65). As shown in Table 5, these correlations were all statistically significant. These findings support the convergent validity of the PROST for assessing disability. Correlation with EQ-5D was weak or very weak (r_s_ = −0.29 to 0.05) and not statistically significant except for the 1-year follow-up visit. This in general supports the discriminant validity of the PROST for assessing HRQoL. Finally, no consistent pattern was observed between PROST and Pain NRS scores.

**Table 5.** Construct Validity AO Spine PROST: Convergent and Discriminant Validity Based on Correlations With ODI, Pain NRS and EQ-5d

Timepoint	Instrument	r_s_	*P*-value
Baseline	ODI	−0.67	<.001
	Pain NRS	−0.27	.012
	EQ-5D	−0.06	.580
6-week	Pain NRS	−0.58	<.001
	EQ-5D	0.02	.876
3-month	ODI	−0.75	<.001
	Pain NRS	−0.57	<.001
	EQ-5D	−0.03	.781
6-month	Pain NRS	−0.64	<.001
	EQ-5D	−0.07	.559
1-year	ODI	−0.84	<.001
	Pain NRS	−0.70	<.001
	EQ-5D	−0.29	.014
2-year	ODI	−0.89	<.001
	Pain NRS	−0.69	<.001
	EQ-5D	0.05	.703
Change between baseline and 1-year	ODI	−0.65	<.001
	Pain NRS	−0.40	.001
	EQ-5D	−0.20	.088

Abbreviations: r_s_: Pearson correlation

### Responsiveness

Responsiveness was assessed by comparing PROST scores from baseline through all follow-up time points up to 2 years post-injury. Mean and median PROST scores for the different timepoints showed gradual increasing over time and changes at each follow-up visit compared to baseline were significant. Effect size (ES) and standardized response mean (SRM) from baseline to 2-year follow-up are shown in Table 6. They were above one at all timepoints and demonstrate that the PROST is highly responsive and capable of detecting substantial clinical improvements over time.

**Table 6.** Responsiveness AO Spine PROST From Baseline Up to 2-Year Follow-Up With Effect Size and Standardized Response Mean

Timepoints	Mean (SD)	Change (SD)^*^	ES	SRM
Baseline	37.8 (16.3)			
6-week	63.1 (15.8)	25.3 (16.6)	1.5	1.5
3-month	74.5 (14.3)	36.8 (15.9)	2.3	2.3
6-month	82.7 (13.5)	44.9 (16.6)	2.7	2.7
1-year	86.9 (12.4)	49.1 (16.7)	3	2.9
2-year	89.6 (12.4)	51.8 (16.5)	3.2	3.1

Abbreviations: SD, standard deviation; ES, effect size; SRM, standardized response mean.

^*^P-value <.001.

## Discussion

This study investigated the Minimal Clinically Important Difference (MCID) of the AO Spine PROST (Patient Reported Outcome Spine Trauma). The MCID for a moderate change in the PROST score was 10.6, based on the distribution-based methodology used in the current study. Additionally, this study represents the first long-term, prospective validation of the PROST with follow-up extending up to 2 years post-trauma in an international cohort. The findings demonstrate that the PROST has strong psychometric properties and high responsiveness over time.

The concept of the MCID was introduced by Jaeschke et al in 1989 to assess whether differences in treatment effects are meaningful from the perspective of individuals living with a given condition.^23^ They defined MCID as ‘the smallest difference in score, within the domain of interest, which patients perceive as beneficial and which would mandate, in the absence of troublesome side effects and excessive costs, a change in the patient’s management'. In short, this definition centers the patient perspective and reflects the smallest difference that people living with a specific condition perceive as beneficial. Methodological approaches for determining the MCID are generally categorized into 2 main types: distribution-based and anchor-based methods.^24,25^ Distribution-based methods assess the meaningfulness of change by comparing observed score differences to statistical properties of the sample, such as the standard deviation or standard error of measurement.^18^ In contrast, anchor-based approaches link changes in the outcome measure to an anchor that is defined as another measure of change. Most commonly, this comparison is done with an external question, defined on a Likert scale.^24,26^ The selection of an appropriate anchor is not solely statistical; it often reflects clinical judgment, patient input, or expert consensus regarding what constitutes a meaningful change.^27^ In the current study, a distribution-based method was employed due to the lack of an appropriate anchor within the available dataset. This approach is widely accepted, and prior literature suggests that a moderate effect size (0.5 SD) is a reasonable approximation of the MCID.^18^ Moreover, it is widely recommended that MCIDs derived through anchor-based methods be supported or validated using distribution-based analyses to enhance interpretability and robustness.^28^ Based on this approach, the estimated MCID for the PROST score was 10.6 points. From a clinical perspective, a change of this magnitude can be interpreted as a meaningful change in patient-reported function, reflecting a noticeable improvement or deterioration in overall function as related to their spine injury, and considered clinically important by treating clinicians.

To the best of our knowledge, no MCID thresholds have been established in spine trauma populations for other instruments used in this study, that is, ODI and EQ-5D. This is not surprising as those measures where not developed and validated for spine trauma patients, rather for degenerative lumbar conditions and the generic population, respectively.^29^ Consequently, the constructs assessed by these instruments only partially overlap with those captured by the PROST, which was specifically designed to assess functional outcomes following spine trauma. This difference in scope may explain the relatively weak correlations observed with the EQ-5D. Concerning the Pain NRS scale, expert consensus has proposed that a 30% reduction in self-reported pain represents a clinically meaningful improvement, particularly in chronic pain conditions, including those following spinal cord injury.^30,31^ However, pain intensity represents only one component of recovery after spine trauma, which may contribute to the relatively weak correlation between the PROST and Pain NRS. To date, the current study is also the first to prospectively evaluate the long-term validity of the PROST with structured follow-up up to 2 years. A previous study by Buijs et al did investigate the long-term validity of the PROST, with a median duration of follow-up being 94.5 months, however they did not perform a prospective follow-up, rather a cross-sectional long-term assessment of the PROST together with other questionnaires.^32^ Nonetheless, they also found very good long-term validity results for the PROST. Similarly, earlier research demonstrated excellent responsiveness of the PROST in a 3-month follow-up, with effect size (ES) and standardized response mean (SRM) values of 1.81 and 2.03, respectively. In the present study, these values were even higher at 3 months (ES and SRM = 2.3) and continued to increase at 2 years (ES = 3.2, SRM = 3.1), indicating strong responsiveness of the instrument across time.

This study found good psychometric properties for the PROST. Interestingly, the item-total correlation analysis identified ‘Urinating’, ‘Bowel movement’ and ‘Sleep’ as having the weakest correlations with the total score. This is likely due to the inclusion of only neurologically intact patients in this study. Bladder and bowel dysfunctions may be major impairments in patients with severe neurological deficit.^33^ Similar findings were reported in the initial PROST development phase and in the Dutch and English validation studies, which also focused on patients with no, transient or mild neurological deficit.^8,9^ Ongoing validation efforts now include individuals with motor-complete spinal cord injury, thereby broadening the instrument’s applicability across the full spectrum of spinal trauma.^34,35^ Beyond the aforementioned validation studies for the Dutch and English versions, the PROST has since been validated in several other languages, including German, Slovak, Nepalese, and most recently Finnish.^36-39^ Given the increasing adoption of the PROST as an outcome measure in clinical research, the establishment of its MCID represents a critical advancement in facilitating the meaningful interpretation and application of its scores.^40-46^ The identified MCID can help distinguish statistically significant changes from those that are meaningful to patients. In clinical research, this threshold may be used to determine the proportion of patients achieving a clinically meaningful improvement following different treatment strategies. In routine clinical practice, the MCID may also assist clinicians in monitoring patient recovery over time and evaluating whether observed changes in PROST scores reflect meaningful improvements in functional status.

We do recognize this study has several limitations. First, the patient sample was restricted to those with thoracolumbar burst fractures. However, this subgroup is highly relevant as being most controversial in terms of optimal management, thus making it particularly important to define an MCID for this specific population. Nevertheless, future studies should explore whether similar MCID thresholds apply to broader spine trauma populations as that the identified MCID may not be directly generalizable to patients with other spinal injury types. Second, only Dutch- and English-speaking participants were included as these were the only validated language versions available at the study’s initiation. Although this limitation may somewhat restrict the cultural and linguistic generalizability of the findings, additional translations of the PROST have since become available. Future studies can broaden its scope to include larger patient samples with more diverse linguistic populations and a wider range of fracture types. Finally, the study did not include test-retest reliability assessment, primarily to avoid overburdening participants with additional questionnaires. Nonetheless, previous studies have consistently demonstrated excellent test-retest reliability for the PROST.

In conclusion, this study established the Minimal Clinically Important Difference (MCID) of the AO Spine PROST (Patient Reported Outcome Spine Trauma) using a distribution-based approach, identifying a threshold of 10.6 points as indicative of a moderate clinically meaningful change. Additionally, the instrument demonstrated satisfactory psychometric performance in a prospective, long-term validation with follow-up extending to 2 years post-trauma. These findings further support the PROST as a valid and responsive outcome measure, enhancing its utility for both clinical research and routine clinical practice in spine trauma care.
